# Detection of Cervical Lesion Cell/Clumps Based on Adaptive Feature Extraction

**DOI:** 10.3390/bioengineering11070686

**Published:** 2024-07-05

**Authors:** Gang Li, Xingguang Li, Yuting Wang, Shu Gong, Yanting Yang, Chuanyun Xu

**Affiliations:** 1 School of Artificial Intelligence, Chongqing University of Technology, Chongqing 401135, China; ligang@cqut.edu.cn (G.L.); lxg@stu.cqut.edu.cn (X.L.); yyt123@stu.cqut.edu.cn (Y.Y.); 2 Department of Gastroenterology, Children’s Hospital of Chongqing Medical University, Chongqing 400014, China; chloe.gong@hotmail.com; 3 National Clinical Research Center for Child Health and Disorders, Chongqing 400014, China; 4 Ministry of Education Key Laboratory of Child Development and Disorders, Chongqing 400014, China; 5 Chongqing Key Laboratory of Child Neurodevelopment and Cognitive Disorders, Chongqing 400014, China; 6 College of Computer and Information Science, Chongqing Normal University, Chongqing 401331, China

**Keywords:** cervical cancer, target detection, bounding box loss, adaptive feature extraction

## Abstract

Automated detection of cervical lesion cell/clumps in cervical cytological images is essential for computer-aided diagnosis. In this task, the shape and size of the lesion cell/clumps appeared to vary considerably, reducing the detection performance of cervical lesion cell/clumps. To address the issue, we propose an adaptive feature extraction network for cervical lesion cell/clumps detection, called AFE-Net. Specifically, we propose the adaptive module to acquire the features of cervical lesion cell/clumps, while introducing the global bias mechanism to acquire the global average information, aiming at combining the adaptive features with the global information to improve the representation of the target features in the model, and thus enhance the detection performance of the model. Furthermore, we analyze the results of the popular bounding box loss on the model and propose the new bounding box loss tendency-IoU (TIoU). Finally, the network achieves the mean Average Precision (mAP) of 64.8% on the CDetector dataset, with 30.7 million parameters. Compared with YOLOv7 of 62.6% and 34.8M, the model improved mAP by 2.2% and reduced the number of parameters by 11.8%.

## 1. Introduction

Cervical cancer ranks as the fourth most common cancer globally, posing a significant threat to female health. In 2020, there were 604,127 new cases of cervical cancer worldwide, with 341,831 deaths attributed to the disease. Alarmingly, 85–90% of these new cases occurred in developing countries [1]. Research has found that cervical cancer is almost entirely preventable due to its long latency period. Research indicates that persistent infection with high-risk types of human papillomavirus (HPV) is the primary cause of cervical cancer. In recent years, HPV vaccination has been shown to effectively reduce cervical diseases caused by HPV. However, widespread HPV vaccination is limited due to various economic and policy differences among countries. Therefore, the thinprep cytologic test (TCT) is essential. In medicine, TCT is an effective method for preventing cervical cancer. This method requires pathologists to prepare and stain shed cervical cells, and finally, under a microscope, to make preliminary diagnostic results based on subtle differences in morphology and structure between cervical lesion cells and normal cells. However, detecting thousands of cells in cytological images with gigapixel resolution is extremely time-consuming and prone to errors, requiring pathologists to possess specialized knowledge and extensive experience.

In recent years, the rapid development of deep learning in the field of imaging has enabled artificial intelligence to achieve good results in many medical tasks, including the classification of skin malignancies [2], the classification of lung cancer [3], and the detection and classification of retinal diseases [4]. In screening for cervical cancer, artificial intelligence can help address the shortage of pathologists and effectively improve the efficiency of cervical cancer diagnosis. Early detection methods for cervical lesion cells [5,6,7] usually include candidate region segmentation, feature extraction, and classification. However, the detection performance of this method relies on the accuracy of segmentation and the effectiveness of hand-crafted features. In conjunction with deep learning models, several convolutional neural networks-based methods [8,9,10,11,12,13] have emerged for cervical lesion cell classification. Although these methods have improved the performance of computer-aided diagnosis, numerous overlapping and adherent cells in cell images make it difficult to achieve the required segmentation accuracy, limiting the identification of cervical lesion cells.

The important goal of screening is to find diseased cells in cervical cell images and recognize them in different lesion stages. As a result, methods have emerged to detect lesion cell/clumps directly from cervical cell images in an end-to-end manner using target detection models [14,15,16,17,18,19,20,21]. In [14], Faster-RCNN [22] and RetinaNet [23] were used directly for the detection of large cervical cell datasets. Comparison detectors [15] are used to deal with issues with limited data sets. Yi et al. [16] proposed a dense cascaded regional convolutional neural network with three modules of data augmentation, training set balancing, and dense cascaded R-CNN to enhance the performance of cervical cell detection. A specific YOLOv3 [24] detector was integrated into [19] for cervical cell detection. However, all these methods utilize only local features of the cervical cell images. In this regard, Liang et al. [17] constructed a global context-aware framework to reduce false-positive predictions through image classification branching and weighted loss. Enhancing the region of interest features by exploring the context in [20] improves the detection accuracy.

Despite some notable achievements made by these methods, they have not fully exploited the characteristics of cervical lesion cells in cervical cell images. Cao et. [18] introduced an attention feature pyramid network, which utilizes attention modules to enhance or suppress feature learning, thereby improving detection accuracy. Chen et al. [21] divided the same type into individual cells and clumps through task decomposition, thereby enhancing the ability of model to learn features of a single category. Unlike these methods, our approach employs adaptive feature extraction for cervical lesion cell/clumps, integrating global information from cervical cell images while extracting features of cell/clumps of various shapes and sizes, thereby enhancing the feature representation of cervical lesion cell/clumps.

Adaptive methods are primarily derived from deformable convolutions [25,26,27], which achieve adaptive feature extraction by altering sampling positions with additional offsets. In recent years, many methods have extended deformable convolutions to obtain better feature representations, such as conditional parameterized convolutions [28], dynamic convolutions [29], and decoupled dynamic filtering networks [30]. In medical imaging, adaptive methods hold tremendous potential. Gao et al. [31] embedded adaptive feature extraction methods into U-Net for liver tumor segmentation. An adaptive feature extraction method has been used for COVID-19 X-ray image classification [32].

The bounding box loss plays a crucial role in object detection tasks. In object detection tasks, IoU is commonly used as the bounding box loss, which calculates the intersection over union ratio between the predicted box and the ground truth box. This effectively reflects the degree of overlap between the predicted and ground truth boxes, accelerating model convergence. However, IoU loss also has two drawbacks. Firstly, when the predicted box and the ground truth box do not intersect, IoU is always 0, leading to vanishing gradients during backpropagation. Secondly, when two predicted boxes have the same IoU loss with the ground truth box, it cannot reflect which predicted box is better. Subsequent researchers proposed enhancing model performance by incorporating geometric factors of bounding boxes into penalty terms to address the drawbacks of IoU [33,34,35].GIoU [33] uses the minimum enclosing box to construct the penalty term. DIoU [34] utilizes the normalized center distance between the predicted box and the target box to construct the penalty term. CIoU [34] adds the aspect ratio of the predicted box and the target box to the penalty term based on DIoU. SIoU [35] constructs the penalty term by considering angle cost, distance cost, and aspect ratio. In [36], it is argued that a good loss function should attenuate the penalty of geometric factors when the predicted box overlaps with the ground truth box. Less intervention during training enables the model to achieve better generalization capabilities. In response to this, WIoU [36] is proposed as the bounding box loss. WIoU [36] scales the IoU loss through dynamic focusing and attention distance, reducing the impact of outlier anchor boxes. Considering the effect of bounding box loss on the accuracy of cervical lesion cell detection, We introduce angle attention into WIoU for metric evaluation, termed TIoU, as detailed in Section 2.3.

**Figure 1:**
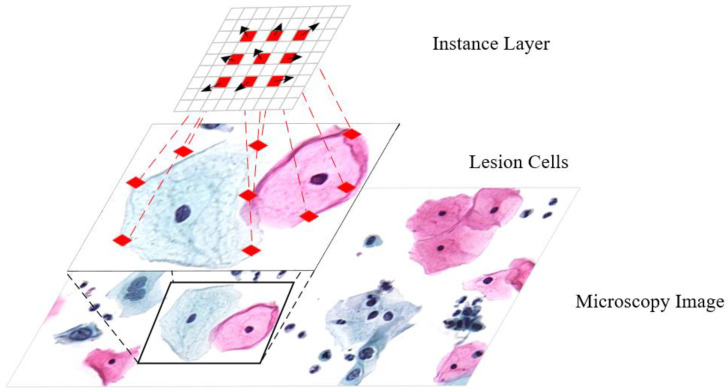
Adaptive feature extraction. Regular convolutions extract features only from specific regions. Deformable convolutions have the ability of geometric transformation, but the transformation ability is limited. Dilated deformable convolutions can adjust the size of dilation to enhance the deformation capability.

We propose an adaptive feature extraction network (AFE-Net) for cervical lesion cell/clumps detection, as shown in Figure 1. Specifically, we propose the Global adaptive bias module (GABM), which includes the adaptive module (AM) with dilated and the global bias mechanism (GBM). That is, the adaptive module acquires adaptive features of cervical lesion cell/clumps while introducing the global bias mechanism to obtain global average information, and then combines the adaptive features with global information. This approach allows our network to better learn the features of each lesion cell/clumps, thereby improving the detection performance of the model. Additionally, we analyze the impact of popular bounding box losses on the model and propose a new bounding box loss, tendency-IoU (TIoU), to enhance the detection accuracy of AFE-Net.

In summary, the contribution of this paper is as follows:

(1) We propose an adaptive feature extraction network, named AFE-Net, for detecting cervical lesion cell/clumps.
(2) Through the adaptive module (AM) and the global bias mechanism (GBM), we divide feature extraction into adaptive feature and global average information extraction, enhancing the ability of the network to extract various shape and size features of cervical lesion cell/clumps.
(3) We discuss the influence of mainstream bounding box losses on cervical lesion cell/clumps detection and propose a new bounding box loss, tendency-IoU (TIoU), to improve the detection accuracy of the model.
(4) Using AFE-Net, we achieve the highest mAP (64.8%) on cervical cell datasets Comparison Detector (CDetector), with a reduction of 11.8% in model parameters compared to the baseline model.

## 2. Materials and Methods

We address the issues encountered in detecting cervical lesion cell images and propose a method based on an adaptive feature extraction network (AFE-Net) built upon YOLOv7 [37]. To tackle the problem of overlapping cell shapes and significant variations in size, leading to lower detection accuracy, we introduce an adaptive feature extraction approach that effectively captures features of cervical lesion cells. Furthermore, considering the global impact of cervical cell images, we propose a global bias mechanism to acquire global average information. Finally, we analyze the impact of different bounding box losses on detection accuracy and propose TIoU loss.

### 2.1. Network Structure

**Figure 2:**
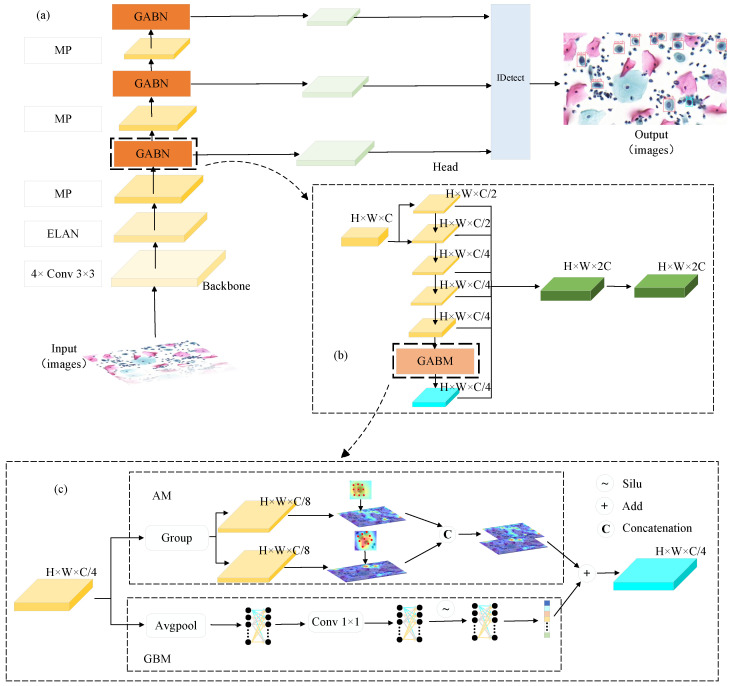
Block diagram of AFE-Net. (**a**) AFE-Net overall structure diagram. (**b**) GABN structure diagram. (**c**) GABM structure diagram with the adaptive module (AM) in the upper half and the global bias mechanism (GBM) in the lower half.

Figure 2a depicts the structural framework of AFE-Net. It mainly consists of three parts: the input end, the backbone network, and the detection head. Effective preprocessing methods are provided at the input end, such as the Mosaic high-order data augmentation strategy, adaptive image adjustment strategy, and multi-scale training, to help improve model accuracy. The backbone network extracts features from input images, comprising an efficient layer aggregation network, MP structure, and global adaptive bias network (GABN). The efficient layer aggregation network enhances feature learning from different features by adding feature bases through residual and grouped convolution methods. GABN primarily consists of a connection-based model scaling network and GABM, as shown in Figure 2b. The connection-based model scaling network utilizes residual connections and channel reduction methods to obtain multi-scale features. The specific details of GABM are in Section 2.2. The detection head adopts the scheme of the base model.

### 2.2. Global Adaptive Bias Network

To address the challenges in cervical cell image detection, we designed a global adaptive bias network, which primarily utilizes a connection-based model scaling network and GABM. The connection-based model scaling network is constructed using numerous residual structures and methods for reducing channels. GABM consists of AM and GBM, as illustrated in Figure 2c. In the design of GABM, we not only consider the shape and size of cervical cell/clumps but also take into account the influence of global information.

Adaptive Module (AM): Unlike regular convolutions, we divide the input features into two groups along channels and apply deformable convolutions with different dilation sizes to each group separately [26], in order to obtain adaptive feature maps under different receptive fields. Then, the resulting feature maps are concatenated to obtain adaptive features for cervical lesion cell/clumps.

Given an input $x\in {ℜ}^{C\times H\times W}$, a void size *d* (in this experiment *d* is 6 and 12), and the current pixel $p_{0}$, which is divided into two groups by channel, the input becomes $x\in {ℜ}^{C/2\times H\times W}$, and the deformable convolution can be expressed as:

(1) $$y\left(p_{0}\right)=\sum_{k=1}^{K}w_{k}m_{k}x\left(p_{0}+p_{k}+△p_{k}\right)$$

where *K* denotes the total number of sampling points, *k* denotes the enumerated sampling points. $w_{k}$, $m_{k}$ and $△p_{k}$ denote the weights, modulation scalars, and offsets, respectively, of the *k* location. $p_{k}\in \left\{\left(-1-d,-1-d),(-1-d,0),\ldots ,(1+d,1+d\right)\right\}$ denotes the position of sampling.

Global Bias Mechanism (GBM): In order to incorporate global characteristics into the output features, we designed a global bias mechanism. Firstly, bias is acquired through adaptive average pooling of the input features. Then, it is adjusted through a 1 × 1 convolution, and finally, outputted through an FC layer with the Silu activation function. This approach enables GABM to generate coherent outputs considering global cell image information.

Compared to previous work, we propose GABM. On one hand, it reduces the feature disparity caused by targets at different scales. On the other hand, our grouping operation effectively reduces computational resources.

### 2.3. Bounding Box Loss

To enhance focus on the prediction boxes, this paper constructs the TIoU bounding box loss. The angle cost (Equation (2)) in SIoU is utilized to build angle attention (Equation (3)), where the angle cost is 0 when the center point connects parallel to the x-axis or y-axis. The angle cost is 1 when the center point connects at a 45-degree angle to the x-axis. This angle attention enables bounding boxes to move towards the x-axis or y-axis, reducing the freedom of the boxes. This method allows for a better tendency of bounding box loss even when sharing the same attention.

The mathematical representation of the TIoU loss function is as follows:

(2) $$\Lambda =sin(2sin^{-1}\frac{min(\left|x-x_{gt}\right|,\left|y-y_{gt}\right|)}{\sqrt{{\left(x-x_{gt}\right)}^{2}+{\left(y-y_{gt}\right)}^{2}}})$$

(3) $$\eta =\frac{\Lambda +1}{2}$$

(4) $$L_{TIoU}=\frac{\beta }{\delta \alpha^{\beta -\delta }}e^{\eta \frac{{\left(x-x_{gt}\right)}^{2}+{\left(y-y_{gt}\right)}^{2}}{{\left(W_{g}^{2}+H_{g}^{2}\right)}^{*}}}L_{IoU}$$

(5) $$L_{IoU}=\frac{A\cap B}{A\cup B}$$

**Figure 3:**
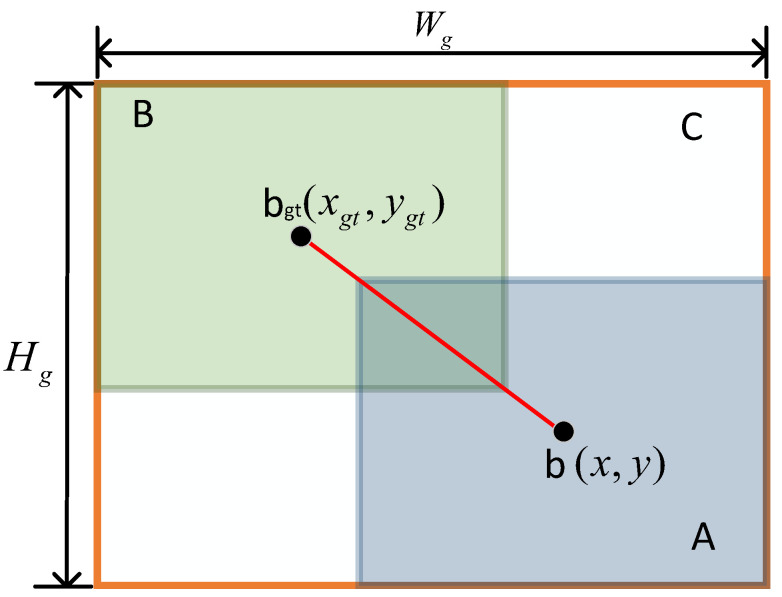
The smallest enclosing box (orange) and the central points’ connection (red). A represents the predicted box. B represents the ground truth box. C represents the minimum closed interval.

In which, *x* and *y* respectively represent the horizontal and vertical coordinates of the center point of the predicted box. $x_{gt}$ and $y_{gt}$ respectively represent the horizontal and vertical coordinates of the center point of the ground truth box. Λ represents the angle cost, while η represents the angle attention. *A* and *B* respectively represent the predicted bounding box and the ground truth bounding box. $W_{gt}$ and $H_{gt}$ respectively represent the width and height of the minimum closed interval, as illustrated in Figure 3. According to [36], α and δ are both constants, with values of 1.9 and 3 respectively, where β represents the abnormality level of the predicted box.

### 2.4. Datasets

**Figure 4:**
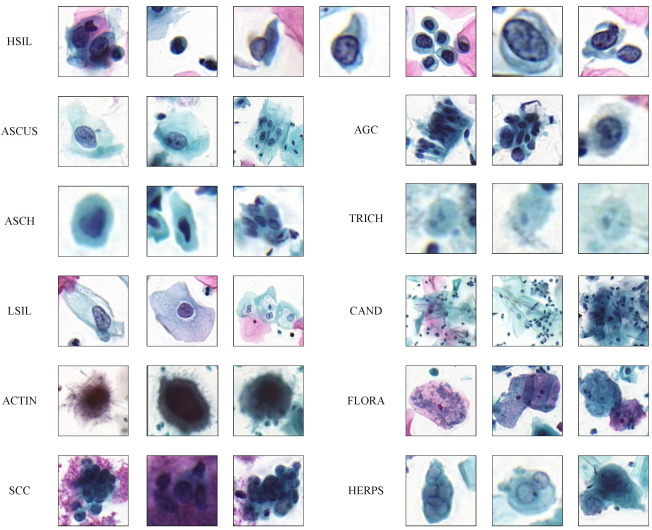
Cervical cell/clumps at different stages of lesions. Cell/clumps within the same category exhibit various features, while different categories share similar characteristics.

In the study of cervical cell analysis using deep learning, common datasets for classification and segmentation tasks include ISBI [38], Sipakmed [39], FNAC [40], and LBC [41]. In contrast, there are fewer datasets available for detection tasks, and most of them are private datasets. In this study, we utilized the public dataset CDetector [15] for our experiments. This dataset comprises 7410 microscopic images of cervical lesion cell/clumps cropped from the whole slide images (WSI) obtained from the Pannoramic MIDI II digital slide scanner. The corresponding specimens were prepared using the Papanicolaou staining method. We divided these images into training and testing sets, with 6666 images in the training set and 744 images in the testing set. The dataset includes 11 categories, and some sample images are shown in Figure 4. The distribution of annotation boxes in the experimental data is shown in Table 1.

### 2.5. Experimental Setup

During training and validation, we resize the images to 640 × 640 for input, preprocess them using Mosaic data augmentation [42] (Randomly crop four images and then stitch them together into one image for training data) and multi-scale training (Randomly scale the input image by a factor of 0.5 or 1.5) methods, and initialize the training weights using MS COCO (A large-scale image dataset developed and maintained by Microsoft). We utilize the SGD optimizer with an initial learning rate of 0.01, which is reduced using Cosine annealing. After the 100th epoch, the training stops. Each batch randomly samples 8 images, with weight decay set to 0.0005 and momentum to 0.937.

### 2.6. Evaluation Metrics

This experiment evaluates the performance of various methods using the mean Average Precision from popular object detection competitions such as Pascal VOC and MS COCO. When calculating, the IoU threshold is set to 0.5. The computation process of mAP is as follows:

Step 1: Obtain the Precision (the proportion of true positives among the samples predicted as positive) and Recall (the proportion of samples predicted as positive among the actual positive samples) for each class.

(6) $$Precision=\frac{TP}{TP+FP}$$

(7) $$Recall=\frac{TP}{TP+FN}$$

In the formula, TP represents the true positives, referring to the number of correctly detected cervical lesion cell/clumps. FP represents the false positives, indicating the number of incorrectly detected cervical lesion cell/clumps. FN represents the false negatives, referring to the missed cervical lesion cell/clumps.

Step 2: Plot the AP curve for individual classes using precision and recall, then calculate the Average Precision AP value for each class using the AP formula.

(8) $$AP=\int_{0}^{1}P\left(R\right)dR$$

Step 3: Sum up all the AP values obtained for each class, then calculate the average to obtain the final mAP

(9) $$mAP=\frac{\sum_{i=1}^{m}AP_{i}}{m}$$

In addition to this, this experiment will also use the number of model parameters as a reference.

## 3. Results

### 3.1. Comparison with State-of-the-Art Methods

We compared our proposed AFE-Net with known methods for cervical lesion cell/clumps detection. Table 2 shows the results, from which it can be observed that: (1) Among the object detection models, YOLOv7 demonstrates superior detection performance compared to other models. (2) Based on YOLOv7, our method significantly improves the model’s detection accuracy while reducing the model’s parameter count, indicating the effectiveness of our proposed adaptive feature extraction network and TIoU. (3) Compared to state-of-the-art models, our AFE-Net outperforms other models, demonstrating the superiority of our approach.

**Figure 5:**
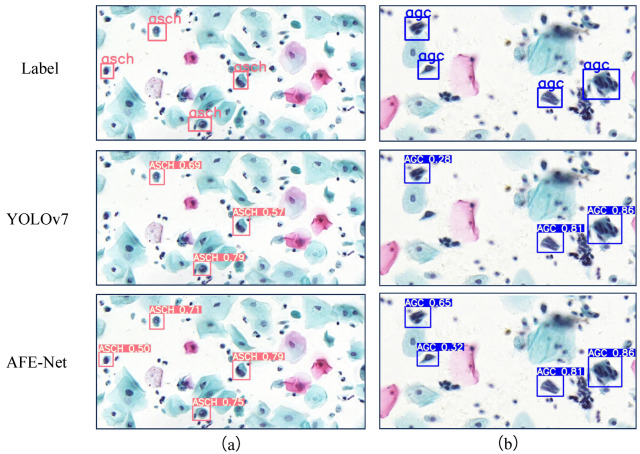
Results of cervical lesion cell/clumps detection by YOLOv7 and AFE-Net. Text boxes of different colors represent cervical cells belonging to different lesion categories. (**a**) Detection of individual lesion cells by YOLOv7 and AFE-Net. (**b**) Detection of lesion clumps by YOLOv7 and AFE-Net.

Furthermore, Figure 5 illustrates some example cases of cervical lesion cell/clumps detection using YOLOv7 and AFE-Net. In (a) and (b), the results for detecting individual lesion cells and clumps are presented, respectively. YOLOv7 exhibits instances of missed detections in both (a) and (b), while AFE-Net does not encounter such issues, demonstrating higher detection accuracy than YOLOv7. Based on this observation, we conclude that our AFE-Net is capable of detecting regions missed by YOLOv7 and exhibits excellent capability in identifying both lesion cells and lesion clumps.

**Figure 6:**
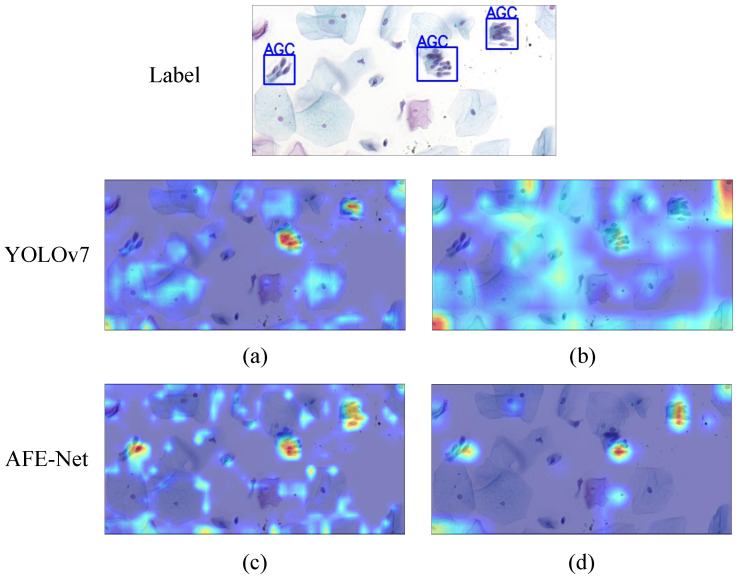
The feature map visualizations of YOLOv7 and AFE-Net, where deeper warm colors indicate higher attention. (**a**,**b**) represent the visualizations of YOLOv7 at Conv37 and Conv50, respectively, while (**c**,**d**) depict AFE-Net at Conv37 and Conv50, respectively.

To demonstrate the effectiveness of GABM, this paper visualizes partial outputs of YOLOv7 and AFE-NET using heatmap visualization. These output feature maps are derived from the Conv37 and Conv50 stages of the backbone. Firstly, the output feature maps of these two stages are summed and averaged along the channel dimension, and then upsampled using the sigmoid function to restore the feature maps to the original image size, as shown in Figure 6. At Conv37, YOLOv7 exhibits good focus on two targets, while AFE-NET shows good focus on all targets, as depicted in Figure 6a and Figure 6c, respectively. However, at Conv50, the attention of YOLOv7 is relatively dispersed, lacking sufficient focus on all targets, whereas the attention of AFE-Net to all targets is more pronounced, as shown in Figure 6b and Figure 6d, respectively. This indicates that when detecting cervical lesion cells using AFE-Net, the model can better focus on the features of cervical lesion cell/clumps.

### 3.2. Ablation Study

#### 3.2.1. Adaptive Feature Extraction Experiments

We conducted relevant experiments on the feature extraction network for cervical lesion cell/clumps. Our experimental settings are as follows: (1) replacing the Efficient Layer Aggregation Network with a connectivity-based model scaling network (V1), (2) adding AM on top of V1 (V2), (3) adding GBM on top of V1 (V3), and (4) adding GABM on top of V1 (V4). The experimental results are shown in Table 3. It can be observed that the proposed GABM achieves better performance without increasing computational parameters. Additionally, the connectivity-based model scaling network is more conducive to feature extraction for cervical cells. On the other hand, networks utilizing AM and GBM demonstrate better performance, validating the effectiveness of these modules in the feature extraction network.

#### 3.2.2. Generalization Experiment

**Figure 7:**
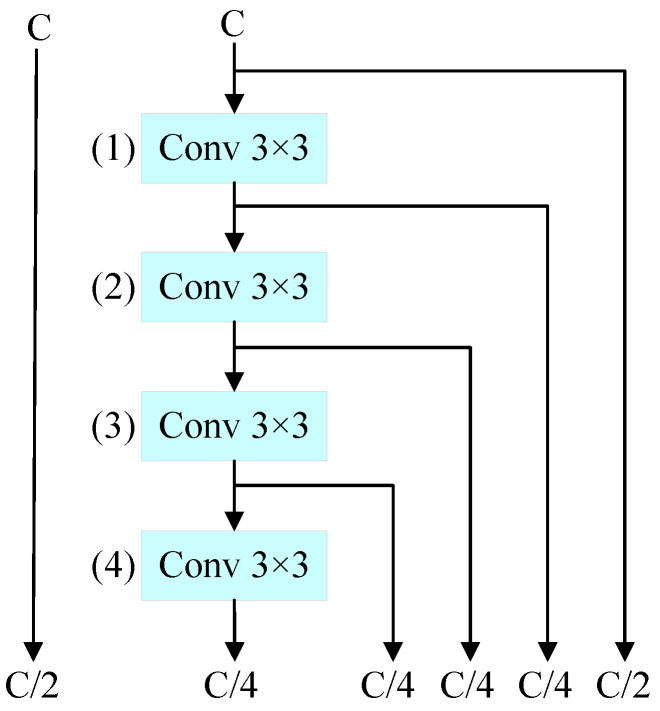
The plan is to replace the convolutions in the connectivity-based model scaling network with GABM. The numbers indicate the positions.

To validate the generality of GABM, we conducted experiments at different positions within the connectivity-based model scaling network. We replaced the 3 × 3 convolutions in the network with GABM. The detailed configurations are shown in Figure 7. From the results in Table 4, it can be observed that the performance of the model improves consistently when using GABM. The largest performance improvement is achieved when replacing the convolution at Figure 7: (4). Therefore, we propose locating GABM at Figure 7: (4).

#### 3.2.3. Bounding Box Loss Experiment

Relevant experiments were conducted on different bounding box losses for cervical lesion cell/clumps. The experimental result is presented in Table 5. The results indicate that replacing the model loss function directly with SIoU [35] and WIoU [36] improves the detection accuracy of the model. When using TIoU as the loss function, the accuracy reaches its peak. Among individual classes, except for the lowest accuracy in the “CAND” category, the results for “ASCUS”, “ASCH”, “AGC”, and “FLORA” all surpass the detection results of SIoU [35] and WIoU [36]. The accuracy for other individual classes lies between SIoU [35] and WIoU [36]. The features of some low-quality examples in the samples are challenging to learn. With the dynamic focusing mechanism reducing the impact of low-quality bounding boxes, focusing on ordinary-quality bounding boxes through angle and distance attention can better improve the accuracy of the model. Therefore, we adopt the TIoU loss function.

### 3.3. Experimental Results on the DCCL Dataset

To further validate the effectiveness of our proposed method, we applied it to the DCCL dataset. This dataset comprises slices from 933 positive cases and 234 normal cases. Samples were prepared using the Papanicolaou staining Thinprep method and scanned by three different digital slide scanners, all magnified at 200 times and cropped into images of 1200 × 2000 pixels. Boundary boxes were annotated by six board-certified pathologists with an average of 8 years of experience. The entire dataset consists of 6301 images, including 3343 images for training, 1193 for validation, and 1765 for testing. The labels in the dataset encompass six lesion types: ASC-US, ASC-H, LSIL, HSIL, SCC, and AGC, along with one false positive label: NILM. The specific distribution of classes is outlined in Table 6.

The comparison between the proposed method and the baseline model is presented in Table 7. It can be observed that AFE-Net achieves higher precision in both fine-grained and coarse-grained detection compared to the baseline. There is a noticeable improvement in the detection accuracy for ASC-US, LSIL, ASC-H, and HSIL. However, the detection accuracy is still not high. This is attributed to the incomplete labeling of the DCCL dataset, making it suitable for semi-supervised learning.

## 4. Discussion

We propose using AFE-Net for the detection of cervical lesion cell/clumps, with several noteworthy points: (1) Our approach involves leveraging the popular one-stage detector YOLOv7 to enhance performance. Subsequent research can select appropriate baseline detectors based on different needs. (2) The adaptive module is an effective method for capturing features of different sizes and shapes. However, in experiments, the adaptive module generally performs well in detecting clusters but less effectively for small cells. Therefore, further research is needed on how to more precisely capture adaptive features. (3) Considering the impact of bounding box loss on the detection results of cervical lesion cell/clumps, the TIoU loss function is a method to improve detection accuracy. While considering generalization ability, exploring factors such as angle and center distance is worth further investigation.

## 5. Conclusions

In this paper, we propose AFE-Net for the detection of cervical lesion cell/clumps. Addressing the issue of significant variations in the appearance of cervical lesion cell/clumps of the same lesion type in cervical cytology images, we introduce the adaptive module (AM) for adaptive feature extraction. Simultaneously considering the influence of global information, we employ the global bias module (GBM) to capture global average information. By combining adaptive features with global average information, we enhance the model’s ability to extract features of cervical lesion cells, thereby improving detection performance. Additionally, we analyze the impact of popular bounding box losses on the model and propose the TIoU loss to enhance detection accuracy. Finally, experimental results on public datasets demonstrate that our method achieves good detection performance with a relatively small number of parameters.

## Figures and Tables

**Table 1:** The distribution of annotation boxes for different lesion cells.

Lesion Type	Train	Test	Total
ASCUS	1835	222	2057
ASCH	3891	410	4301
HSIL	26,305	2823	29,128
LSIL	1466	173	1639
ACTIN	144	18	162
SCC	1991	229	2290
AGC	4989	668	5657
TRICH	4977	481	5458
CAND	336	27	363
FLORA	127	24	151
HERPS	272	37	309
total	46,333	5112	51,445

**Table 2:** The experimental results of AFE-Net compared with state-of-the-art methods.

Method	Parameters	mAP (%)
Faster R-CNN [22]	41.7 M	45.5
RetinaNet [23]	36.3 M	45.2
* Comparison detector [15]	-	48.8
* Faster R-CNN [43]	41.7 M	61.6
YOLOv7 [37]	34.8 M	62.6
AFE-Net (our)	30.7 M	64.8

* Indicates the improved method.

**Table 3:** Experimental results of ablation experiments with adaptation networks.

Method	Parameters	mAP(%)
YOLOv7	34.8 M	62.6
YOLO (V1)	30.7 M	63.1
YOLO+AM (V2)	30.6 M	63.7
YOLO+GBM (V3)	30.4 M	63.6
YOLO+GABM (V4)	30.7 M	64.2

**Table 4:** Experimental results at different positions of GABM.

Position	Parameters	mAP (%)
Figure 7: (1)	30.7 M	63.8
Figure 7: (2)	30.7 M	64.0
Figure 7: (3)	30.7 M	63.5
Figure 7: (4)	30.7 M	64.2

**Table 5:** The experimental results of TIoU compared to mainstream bounding box losses under the baseline model.Values shown in bold indicate the highest values.

	IoU	GIoU	CIoU	SIoU	WIoU	TIoU
ASCUS	52.2	52.0	**52.4**	51.4	49.6	51.9
ASCH	28.5	28.8	29.5	28.8	31.3	**31.6**
LSIL	54.9	59.0	56.0	59.3	**60.8**	60.5
HSIL	58.4	56.9	56.6	**58.8**	58.0	58.6
SCC	**39.8**	31.9	35.6	38.4	37.1	37.8
AGC	**72.6**	72.5	69.7	69.8	71.8	72.3
TRICH	69.0	68.8	**69.8**	68.3	66.6	66.6
CAND	84.2	**92.3**	80.8	85.9	82.3	77.4
FLORA	76.0	67.0	77.4	80.0	76.8	**83.4**
HERPS	83.3	85.4	**86.3**	80.6	84.5	82.8
ACTIN	74.8	70.1	74.7	71.2	**77.8**	74.4
mAP (%)	63.1	62.2	62.6	63.0	63.3	**63.4**

**Table 6:** The class distribution of the DCCL dataset.

Lesion Type	Train	Val	Test	Total
ASC-US	2471	838	1378	4687
ASC-H	1147	543	591	2281
HSIL	5890	1807	3482	11,179
LSIL	1739	356	595	2690
SCC	3006	1225	2731	6962
AGC	122	20	31	173
NILM	2588	1540	2292	6420
total	16,963	6329	11,100	34,392

**Table 7:** Comparison of experimental results between AFE-Net and mainstream detection models on the DCCL dataset.

Method	Fine-Grained							Coarse-Grained
	**mAP**	**ASCUS**	**LSIL**	**ASCH**	**HSIL**	**SCC**	**AGC**	**mAP**
Faster R-CNN [14]	17.1	21.01	20.46	14.1	10.73	10.41	25.71	19.35
Retina-Net [14]	15.93	18.71	19.89	11.86	10.08	12.67	22.39	18.07
Yolov7 [37]	17.29	26.0	16.7	20.8	20.8	10.9	8.56	18.60
AFE-Net	19.02	26.7	21.8	21.7	21.3	9.79	12.8	20.60

## Data Availability

The CDetector dataset is a publicly available dataset that can be found at https://github.com/kuku-sichuan/ComparisonDetector.
